# Integrative system genetic analysis reveals mRNA-lncRNA network associated with mouse spontaneous lung cancer susceptibility

**DOI:** 10.18632/oncotarget.26554

**Published:** 2019-01-08

**Authors:** Yu Zhou, Ming You

**Affiliations:** ^1^ Cancer Center, Medical College of Wisconsin, Milwaukee, WI, 53226, USA

**Keywords:** spontaneous lung cancer susceptibility, lncRNA, co-expression network, system genetic analysis

## Abstract

**Introduction:**

Lung cancer continues to be a significant health burden in the United States. Lung cancer in never smokers is considered as a different disease and underlying mechanism of spontaneous lung cancer susceptibility is still poorly known. Meanwhile, the roles of long non-coding RNAs (lncRNAs), which have multiple functions in biological processes, have seldom been studied in spontaneous lung cancer susceptibility.

**Methods:**

In this study, microarray analyses of normal lung tissues were performed in 23 different mouse strains. LncRNA profile was analyzed by re-annotating exon array for lncRNAs detection. LncRNA/mRNA co-expression networks were constructed and the association between significant lncRNA module and significant mRNA modules was calculated. Finally, Genome-wide association (GWA) results were used to further highlight the key mRNAs and lncRNAs associated with spontaneous lung cancer susceptibility.

**Results:**

Four mRNA modules were significantly associated with spontaneous lung cancer susceptibility. Genes in these modules were enriched in “blood coagulation” and “immune system process”. Only one lncRNA module was significantly associated with spontaneous lung cancer susceptibility. Many lncRNAs in this module were co-expressed with mRNAs in the second most significant mRNA module. This co-expression network contained 113 interactions between 30 lncRNAs and 40 mRNAs. After GWA filtration, two mRNAs (Myo7a and Zfp874a) and two lncRNAs (n290048 and n271850) were highlighted as the candidates responsible for genetic susceptibility to lung cancer.

**Conclusions:**

We firstly used integrative system genetic analysis to report the mRNA-lncRNA network associated with spontaneous lung cancer susceptibility and identified potential targets for lung cancer prevention.

## INTRODUCTION

Lung cancer continues to be a significant health burden in the United States. Over 220,000 new cases are expected in 2017, accounting for about 25% of all cancer diagnoses [1]. The major cause for lung cancers is smoking [2]. However, there are still 15% of lung cancers in men and 53% in women not attributable to smoking [3]. In the last decade, some research groups, including our lab, used inbred mouse model to investigate the mechanism of lung cancers occurring without apparent environmental stimulus (i.e., spontaneous) [4–6]. In these studies, using genome-wide association (GWA) analyses and expression quantitative trait loci (eQTLs) analyses, several key genes or loci were identified to be related to spontaneous lung cancer susceptibility. But since the complexity of spontaneous lung cancer pathogenesis, integrated system genetic approaches are needed to investigate the transcriptome data and further increase the probability of identifying key genes and complex cellular networks.

Long non-coding RNAs (lncRNAs) are defined as RNA transcripts longer than 200 nucleotides without protein-coding function [7]. Accumulating evidence suggests that lncRNAs are involved in various types of molecular mechanisms and many diseases [8]. For examples, lncRNA, HOTAIR (Hox transcript antisense intergenic RNA), was found to be up-regulated in human lung cancer tissues and promote migration and invasion of human lung cancer cell lines [9]. HOTAIR could also promote lung cancer cell metastasis in mice model [9]. The lncRNA MALAT1 (metastasis-associated lung adenocarcinoma transcript 1), was also found to regulate lung cancer metastatic cascade both in human cell lines and in mice model [10]. However, no transcriptome-wide lncRNAs study has been conducted for spontaneous lung cancer susceptibility.

Although some exon microarrays were not designed for lncRNA detection, they contain many probes to expressed sequence tags (ESTs) and prediction-based transcripts. Several studies [11–14] showed that a large portion of these probes can be re-annotated for lncRNAs. For example, Zhou et al. constructed a cancer-related lncRNA database via repurposing microarray probes. Two of the newly identified lncRNAs were confirmed to be associated with prostate cancer cell growth by experiments [15].

In current study, we established a transcriptome-wide lncRNA expression profile by re-annotating Affymetrix Mouse Exon array data from normal lung samples collected from 23 mouse strains. We then constructed mRNA/lncRNA co-expression networks using Weighted Gene Co-expression Network Analysis (WGCNA) and found hub genes based on the gene-module connectivity. Correlations between hub mRNAs and lncRNAs in significant modules were calculated to yield information on transcriptional co-expression in mouse normal lung tissues. In addition, we used GWA analysis results as a filter to pinpoint the most interesting potential regulatory sub-network.

## RESULTS

### Study design and workflow

**Figure 1:**
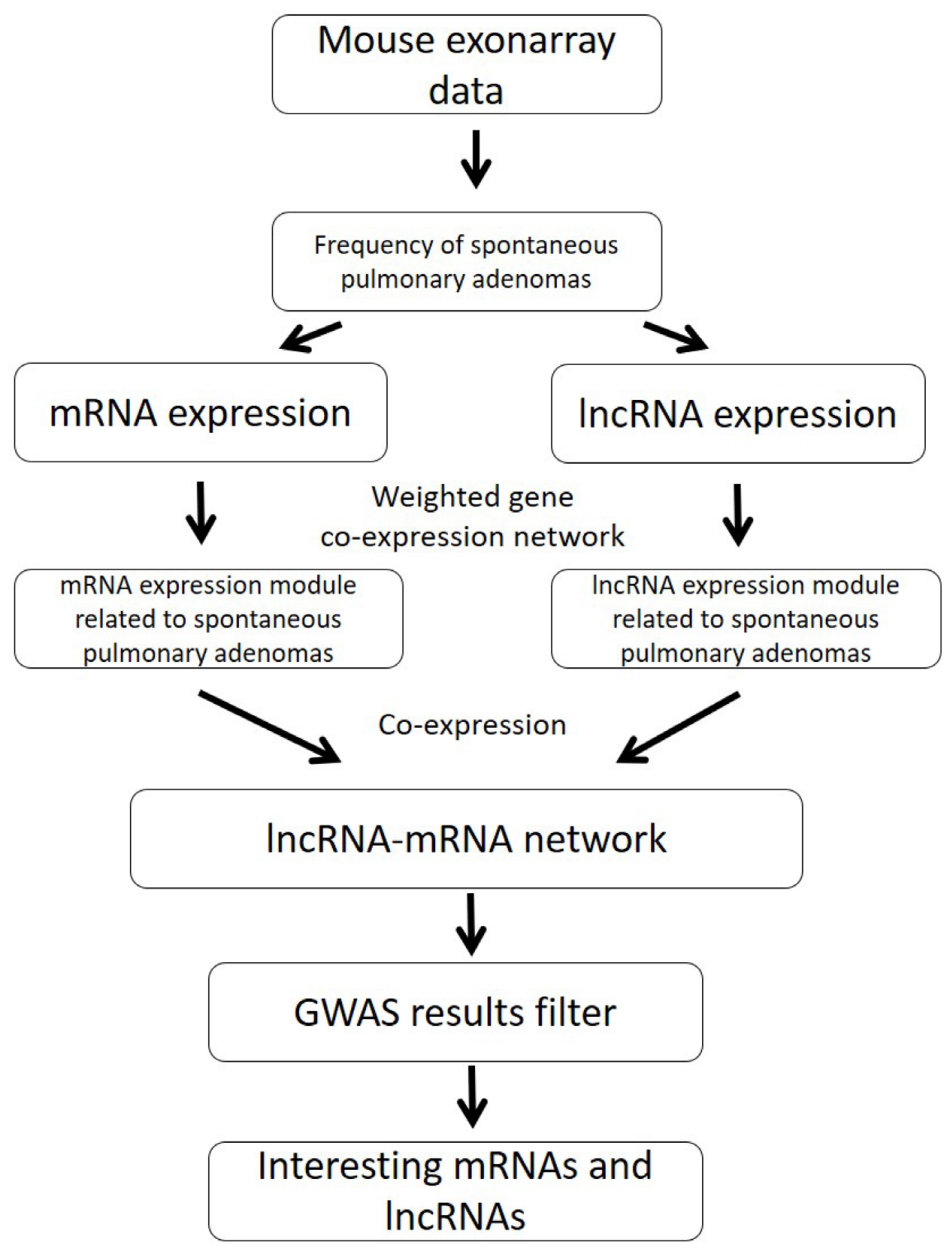
The pipeline of integration analysis to identify mRNA/lncRNA co-expression networks for spontaneous lung cancer susceptibility

In this study, we used RNA expression data of normal lung tissues in different mouse strains to detect key genes for spontaneous lung cancer susceptibility. We annotated RNA expression data to both mRNA and lncRNA profiles. From the co-expression analysis, we aimed to generate potential mRNA-lncRNA network associated with spontaneous lung cancer susceptibility. In Figure 1, we showed our system genetics framework for underlying spontaneous lung cancer pathogenesis. Combining our exon array data and published spontaneous incidence of pulmonary adenomas, system genetics analysis was performed using transcriptome data of lung tissue from 23 mouse strains (total *n* = 138). The detailed strain and spontaneous lung cancer susceptibility information was shown in Table 1.

**Table 1:** The mouse strains and spontaneous lung cancer susceptibility information in this study

Strain	Frequency of spontaneous pulmonary adenomas (^*^100)
129S1/SvMJ	25
A/J	50
BALB/cBYJ	21
BTBRT+tf/J	23
BUB/BnJ	0
C3H/HeJ	0
C57BL/6J	3
C57BR/CD/J	8
C57L/J	10
CBA/J	8
DBA/2J	0
FVB/NJ	23
KK/H1J	0
LP/J	26
NOD/LtJ	6
NON/LtJ	12
NZW/LACJ	0
PL/J	14
PWD/PhJ	9
RIIIs/J	27
SM/J	21
SWR/J	33
WSB/Eij	9

### Construction of mRNA co-expression network and identification of spontaneous lung cancer susceptibility associated modules

**Figure 2:**
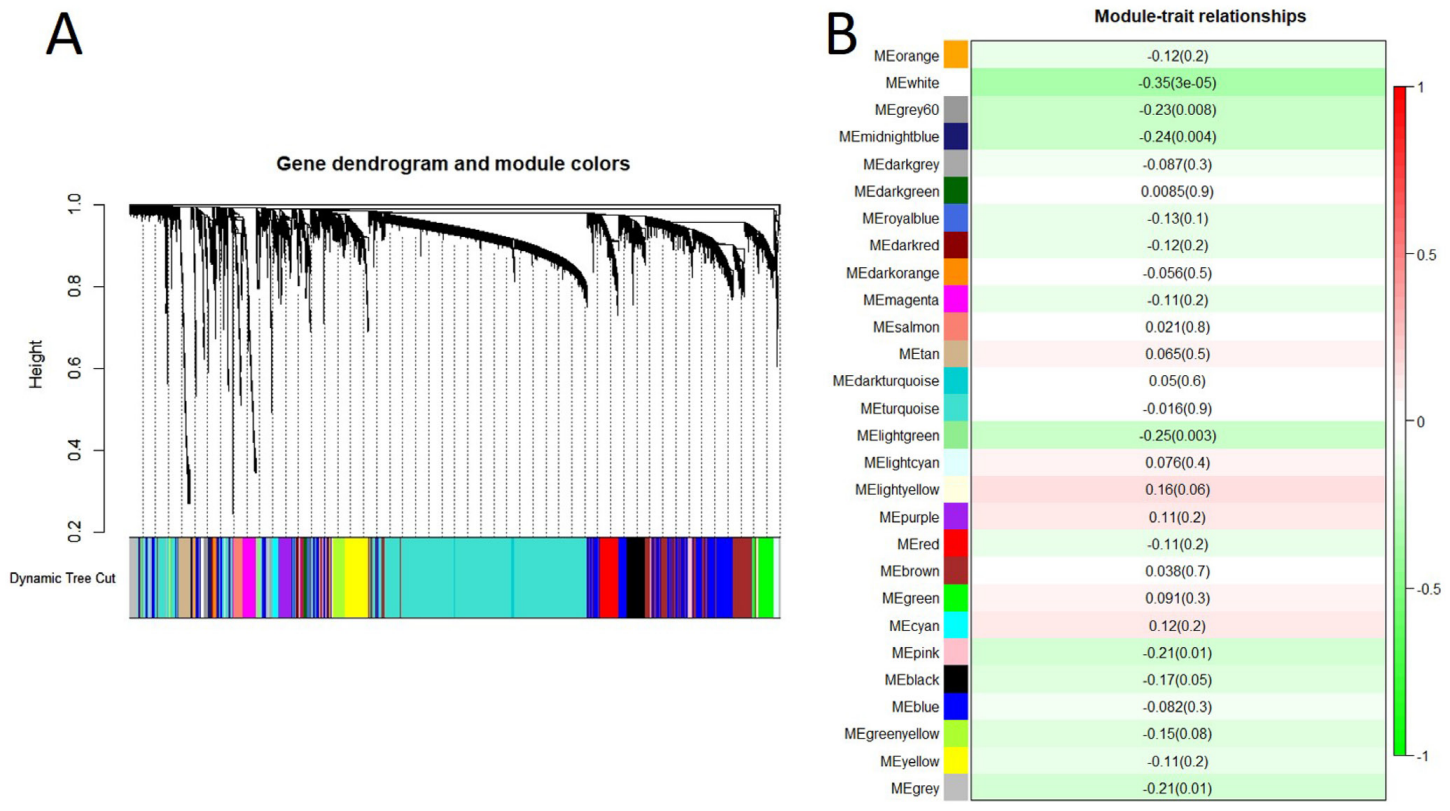
Gene co-expression network analysis of mRNAs by WGCNA (**A**). Hierarchical cluster dendrogram of the 7,500 most variable and connected genes in the lung transcriptome. Modules corresponding to branches are labeled with colors indicated by the color bands underneath the tree. (**B**). Module-trait relationships. Each row corresponded to a module eigengene. Module eigengenes (MEs) were defined as the first principal component calculated using PCA, which can summarize modules’ behavior. Number in each cell shows the correlation of the corresponding module eigengene and spontaneous lung cancer susceptibility, with the *p*-value printed below the correlation. The cell color was coded by correlation according to the color legend.

To investigate the gene-gene interactions important for spontaneous lung cancer susceptibility, after microarray data processing, we selected top 7,500 most variable genes based on variance across the 138 samples and identified 28 modules by WGCNA (Figure 2A). We then examined the biological significance of the identified modules. To test the association of each module with spontaneous lung cancer susceptibility, we applied module eigengenes (MEs) to summarize the behaviors of modules and identified significant association of four modules (white, lightgreen, midnightblue and grey60) with spontaneous lung cancer susceptibility (*p* value < 0.01) (Figure 2B). By Pearson correlation, the ME of each module was negatively correlated with the trait. It meant that all these four modules were negatively correlation with the trait. The white module (module size = 33 genes) showed the most significant results (r = –0.35 and *p* value < 0.001). The top 10 hub genes, module membership (MM) > 0.8, in each module were shown in Table 2.

**Table 2:** Four mRNA modules significantly associated with spontaneous lung cancer susceptibility and top 10 hub genes in each module (ranked by module membership)

Modules	Top 10 hub genes
White	Itga2b, Alox12, Gp9, Slc2a3, Pf4, P2rx1, F5, Ppbp, Itgb3, P2ry12
Lightgreen	Mtmr7, Tmem254a, Myo7a, Casc4, Asah1, Ccdc21, Zfp874a, Bub1b, Art4, Zfp619
Midnightblue	Hcls1, Itgal, Evi2b, Arhgap9, Coro1a, Selplg, Myo1f, Slc11a1, Dok3, Myo1g
Grey60	Slfn4, Dhrs9, Mmp9, Gm11428, Retnlg, Mmp8, Cxcr2, S100a9, Prok2, S100a8

**Figure 3:**
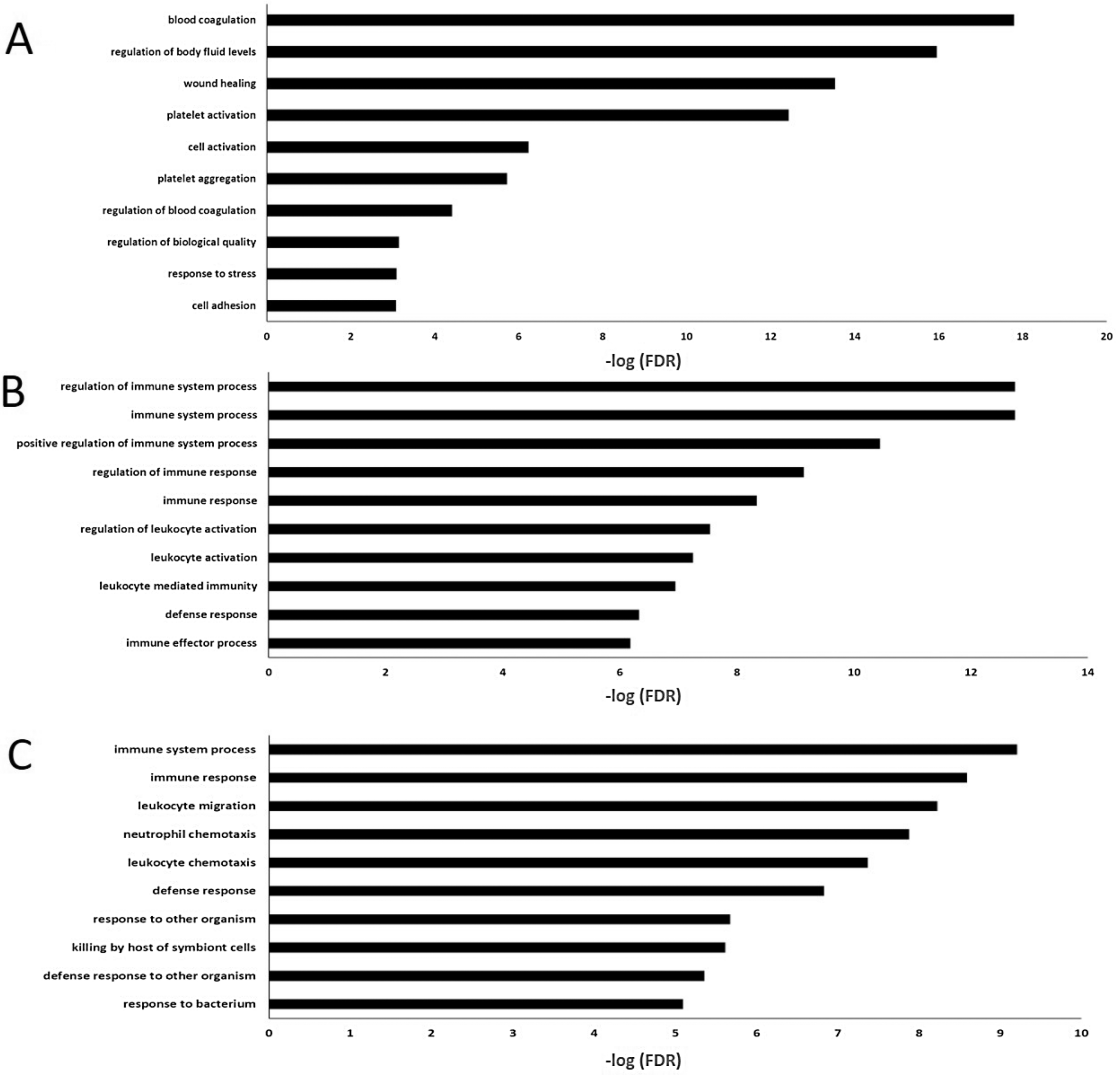
The 10 most enriched GO (Biological process) terms in four significant mRNA modules (No significant enrichment for light green module) (**A**) The 10 most enriched GO terms in white module. (**B**) The 10 most enriched GO terms in midnight module. (**C**) The 10 most enriched GO terms in grey60 module.

**Figure 4:**
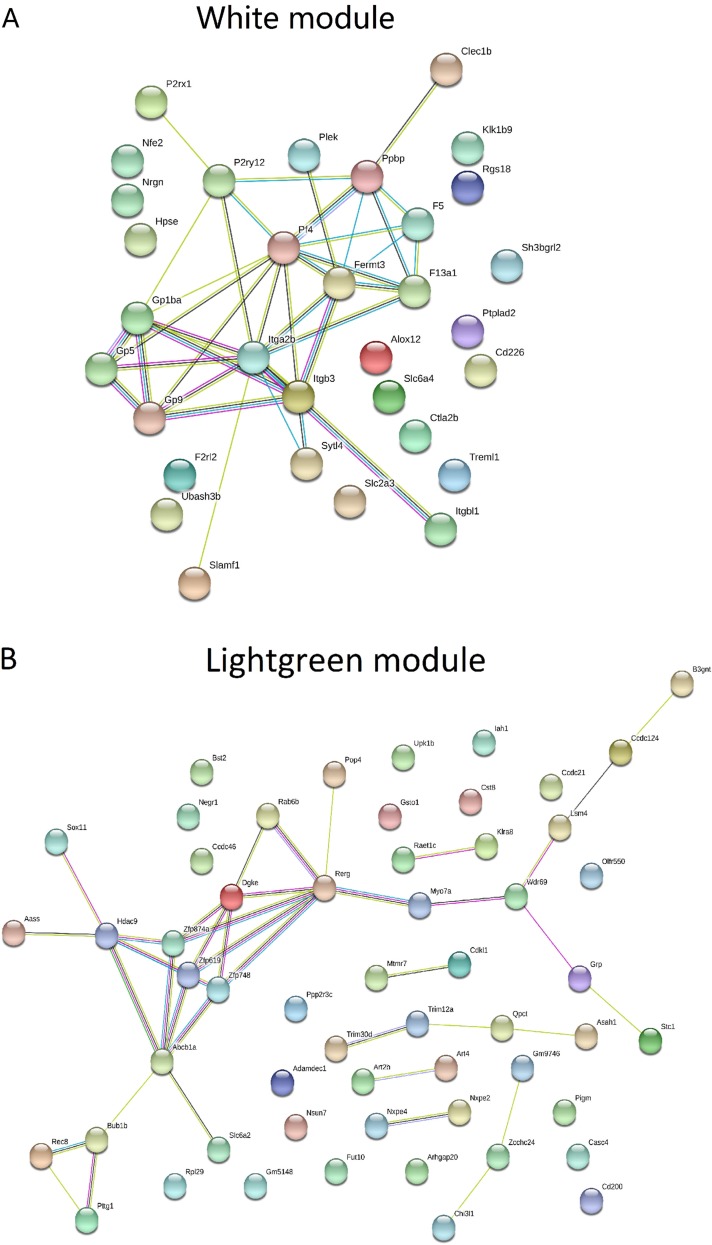

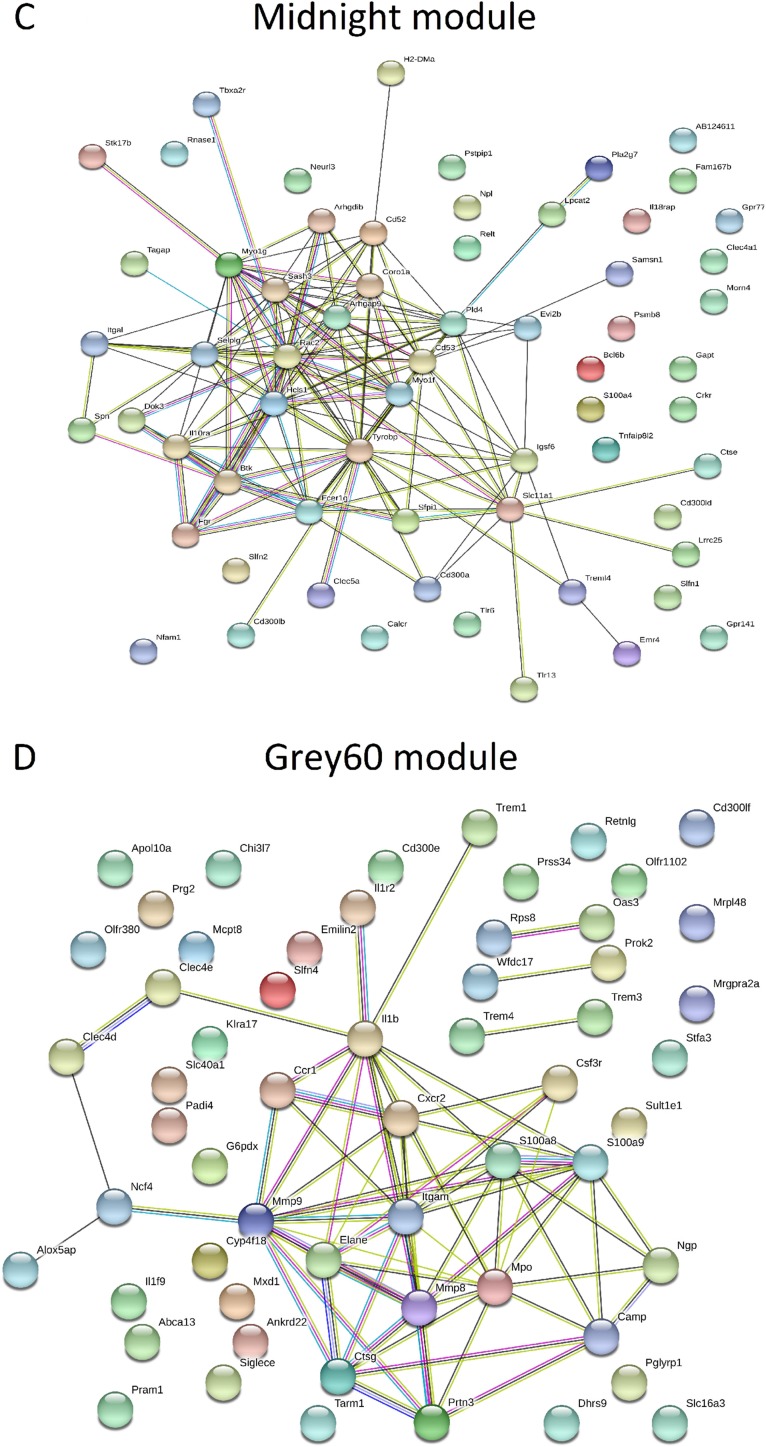
Gene-gene interaction networks in significant mRNA modules The network was built using STRING software and meaning of network edges was based on interaction evidence. (**A**) Genes in white module were enriched in *GO.0007596∼ blood coagulation (FDR < 0.001).* (**B**) The known connections of genes in lightgreen module were poor. No significant gene enrichment was detected. The minimum required interaction score for this module was reduced to 0.15 (default was 0.4) to explore the potential interactions. (**C**, **D**). Genes in midnight and grey60 modules were both enriched in *GO.0002376∼ immune system process (FDR < 0.001).*

Table 3 and Figure 3 summarized the results of enrichment analysis of genes in top four significant modules. Prediction terms with false discovery rate (FDR) less than 0.05 were selected and ranked by FDR values. The most significant gene ontology (GO) term of white module was related to blood coagulation (Figure 3A. FDR < 0.001). In lightgreen module, there was no significant enrichment term. Genes in the midnightblue (Figure 3B) and grey60 modules (Figure 3C) were both enriched in *GO.0002376∼ immune system process.* However, the terms directly related to cancer development, such as cell proliferation, cell cycle and differentiation, were not identified as significant ones. The network of each module was shown in Figure 4.

**Table 3:** Gene enrichment analysis in four mRNA modules significantly associated with spontaneous lung cancer susceptibility

Modules	pathway ID	pathway description	observed gene count	Module size	false discovery rate	matching proteins
White	GO.0007596	blood coagulation	13	33	1.59E-18	F13a1, F2rl2, Fermt3, Gp1ba, Gp5, Gp9, Itga2b, Itgb3, P2rx1, P2ry12, Pf4, Plek, Treml1
Lightgreen	NS.	NS.	NS.	58	NS.	NS.
Midnightblue	GO.0002376	immune system process	27	63	1.76E-13	Btk, Calcr, Cd300a, Cd300lb, Cd300ld, Clec5a, Coro1a, Ctse, Fcer1g, Fgr, Gapt, H2-DMa, Hcls1, Itgal, Myo1f, Myo1g, Nfam1, Pld4, Psmb8, Pstpip1, Selplg, Sfpi1, Slc11a1, Spn, Tlr13, Tnfaip8l2, Tyrobp
Grey60	GO.0002376	immune system process	23	59	6.16E-10	Camp, Ccr1, Cd300lf, Clec4d, Clec4e, Csf3r, Ctsg, Elane, G6pdx, Il1f9, Itgam, Mcpt8, Mmp9, Mpo, Oas3, Padi4, Pglyrp1, Prg2, Prtn3, S100a8, S100a9, Slc40a1, Trem1

### Construction of lncRNA co-expression network and identification of spontaneous lung cancer susceptibility associated modules

**Figure 5:**
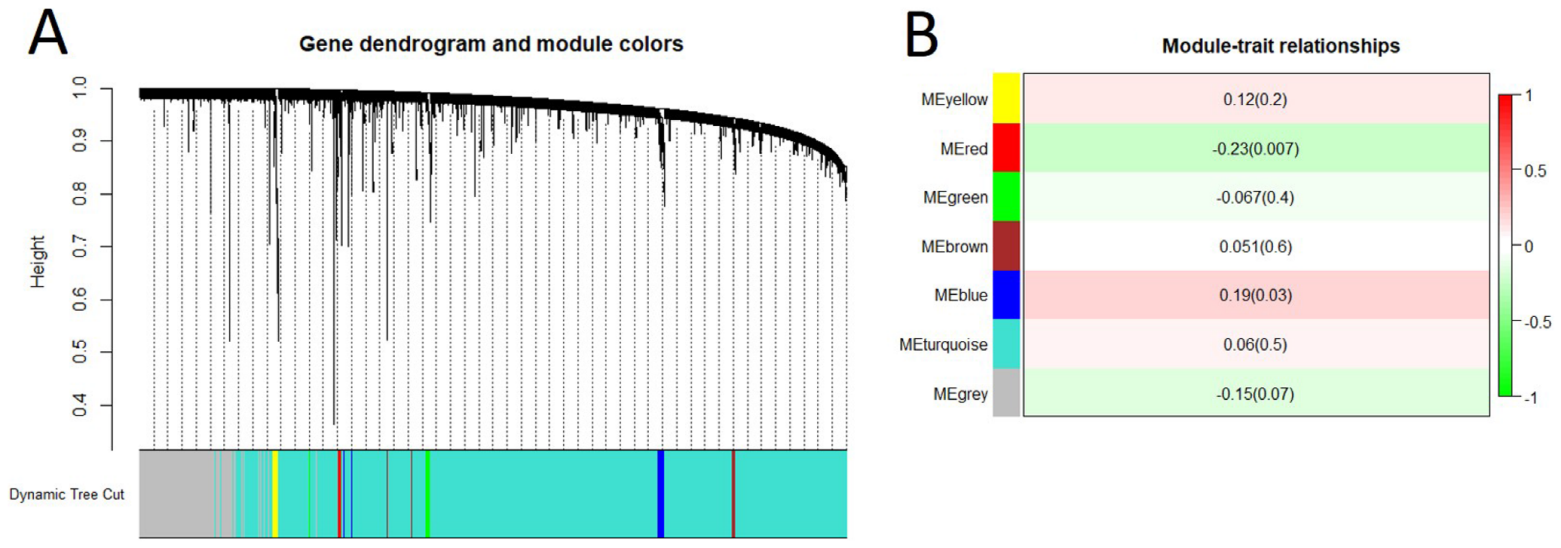
Gene co-expression network analysis of lncRNAs by WGCNA (**A**) Hierarchical cluster dendrogram of the 7,500 most variable and connected lncRNAs in the lung transcriptome. Modules corresponding to branches are labeled with colors indicated by the color bands underneath the tree. (**B**) Module-trait relationships. Each row corresponded to a module eigengene. Module eigengenes (MEs) were defined as the first principal component calculated using PCA, which can summarize modules’ behavior. Number in each cell shows the correlation of the corresponding module eigengene and spontaneous lung cancer susceptibility, with the *p*-value printed below the correlation. The cell color was coded by correlation according to the color legend.

To construct the lncRNA co-expression network, we first re-annotated the probes on the exon array and found a total of 24670 lncRNAs. We then selected 7500 most variable lncRNAs for WGCNA and identified 7 lncRNA modules (Figure 5A). At *p* value less than 0.01, only red module was significantly associated with spontaneous lung cancer susceptibility (Figure 5B).

### mRNA-lncRNA co-expression network

**Figure 6:**
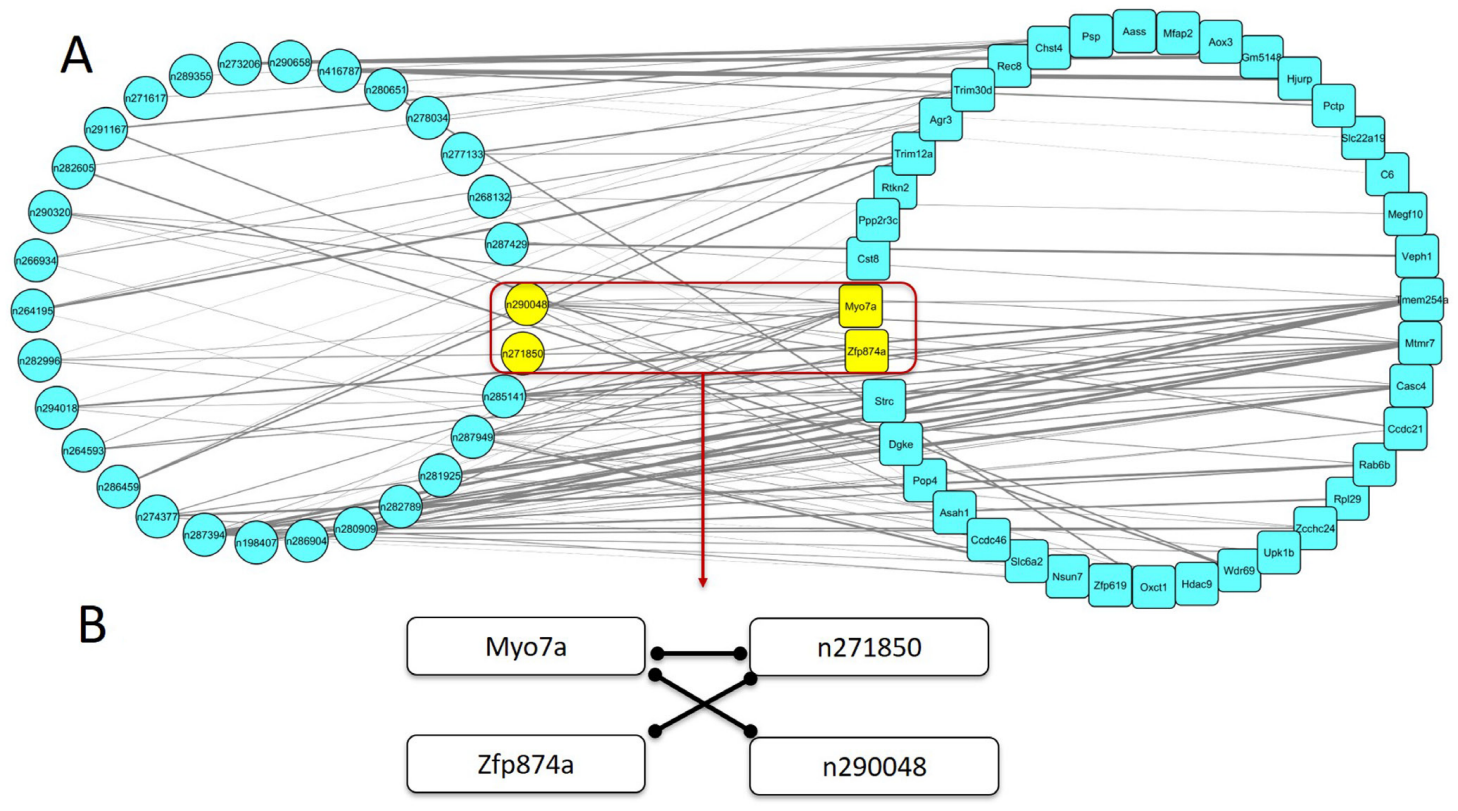
The mRNA-lncRNA co-expression network associated with spontaneous lung cancer susceptibility (**A**) The mRNA-lncRNA co-expression network was constructed among all mRNAs in WGCNA analysis and lncRNAs in top 1 lncRNA module, ranked by the correlation between modules and spontaneous lung cancer susceptibility. The thickness of the edge was based on correlation coefficient between each pair. (**B**). The highlight subnetwork associated spontaneous lung cancer susceptibility filtered by GWA results. Each edge showed that the pair of nodes were co-expressed with each other.

Since one of the major functions of lncRNAs is regulation of gene transcription, we also aimed to study the interactions between mRNAs and lncRNAs. We performed Pearson correlation calculation between lncRNAs in red module and all mRNA involved in WGCNA. Based on the correlation analysis results, we constructed a mRNA-lncRNA co-expression network, including 113 interactions between lncRNAs and mRNAs (*p*- value < 0.05 and absolute value of correlation coefficient > 0.7). The network contained 30 lncRNAs and 40 mRNAs (Figure 6A). The network showed the potential complex regulating relationship between lncRNAs and mRNAs. In the network, all the correlations between lncRNA and mRNA were positive (the absolute values of negative correlation coefficient were less than 0.7) and at least 24 lncRNAs are significantly associated with multiple genes. Among the 40 mRNAs, 26 mRNAs overlapped with genes in mRNA lightgreen module and no overlaps with other three significant mRNA modules.

### Filtration by GWAS results

To further narrow the range of key lncRNAs and mRNAs, we first performed GWAS for spontaneous lung cancer susceptibility. We then applied results to filter the mRNA-lncRNA co-expression network. We used nominal *p* value less than 0.05 as threshold and screened ± 500k nucleotides (nt) around each mRNA or lncRNA in the mRNA-lncRNA co-expression network. If there were risk SNPs locating around a mRNA or lncRNA (± 500k nt), we considered that this mRNA or lncRNA tended to be the key candidate associated with spontaneous lung cancer susceptibility. After filtration, we found that a highlight sub-network including 2 mRNAs and 2 lncRNAs (Table 4, Figure 6B). In this sub-network, expression of Myo7a was positively correlated with expression of lncRNAs n271850 and n290048. And expression of n271850 was also positively correlated with expression of Zfp874a. Ranked by MM, Myo7a and Zfp874a were the third and seventh hub genes in mRNA lightgreen module, respectively. In lncRNA red module, n290048 and n271850 were the 10th and 11th hub genes ranked by MM (Table 5).

**Table 4:** GWAS filtration for each mRNA or lncRNA in the mRNA-lncRNA co-expression network

Nearby genes	Rs#	Alleles	Chromosome	Position	*P* value
lncRNA n290048	mm37-11-113092526	A/T	11	1.13E+08	0.03
lncRNA n271850	mm37-13-67762022	G/C	13	67762022	0.01
Myo7a	mm37-7-105198157	C/T	7	1.05E+08	0.03
Myo7a	mm37-7-105203202	G/A	7	1.05E+08	0.03
Myo7a	mm37-7-105267807	C/G	7	1.05E+08	0.02
Myo7a	mm37-7-105277395	A/G	7	1.05E+08	0.02
Zfp874a	mm37-13-67503131	C/T	13	67503131	0.01

**Table 5:** lncRNAs and mRNAs in the highlighted sub-network

Rank in the modules	Module	Module membership	Chromosome	Start	Stop	Strand	Annotation
3	lightgreen	0.89	chr7	1.05E+08	1.05E+08	-	Myo7a
7	lightgreen	0.83	chr13	6.75E+07	6.76E+07	-	Zfp874a
10	red	0.79	chr11	1.13E+08	1.13E+08	-	n290048
11	red	0.79	chr13	6.78E+07	6.78E+07	-	n271850

## DISCUSSION

Spontaneous lung cancer susceptibility widely varies in different strains of mouse. To identify the candidate genes and network which could predict spontaneous lung cancer susceptibility, in this study, we constructed a co-expression network by microarray expression data from normal lung tissues of 23 different mouse strains. Comparing to traditional reductionist methods that may not be able to explore the overall map of gene interactions, systems genetics approach in this study could provide an unbiased and more comprehensive view of not only the genes involved in cellular function, but also key mRNA-lncRNA interactions. Using WGCNA, we generated 28 modules, each of which contained genes that shared similar expression patterns. Four modules showed significant association with the spontaneous lung cancer susceptibility. Using the connectivity between the genes in same module, we provided an objective filter for rank-ordering genes and found several sets of hub genes. We then elucidated the biological functions of significant modules via gene enrichment analysis. Meanwhile, we profiled the expression of lncRNAs by re-annotating the exon array data and applied WGCNA to construct a lncRNA co-expression network consisted of 7 modules. One of these modules was significantly associated with spontaneous lung cancer susceptibility. Through constructing mRNA-lncRNA co-expression network, we established a framework to map interaction between lncRNAs and mRNAs. To further pinpoint key lncRNAs and mRNAs, we conducted a GWA study and screened the SNPs around mRNAs or lncRNAs in mRNA-lncRNA co-expression network. Two mRNAs (Myo7a and Zfp874a) and two lncRNAs (n290048 and n271850) were highlighted as the most important genes and lncRNAs for spontaneous lung cancer susceptibility prediction. This comprehensive system genetic analysis could provide critical insights into the underlying mechanisms for spontaneous lung cancer susceptibility.

In mRNA WGCNA, the white module showed the most significant correlation with spontaneous lung cancer susceptibility. Through GO analysis, genes in white module were identified to be mainly enriched in blood coagulation. However, there is no evidence showing the direct effect of blood coagulation on spontaneous lung cancer susceptibility. Genes in both midnightblue module and grey60 module were enriched in immune system process. Since the lung tissue used in this study were health and normal, it should not be caused by inflammation in tissues. The lung cancer occurring among non-smokers who never smoked was considered to be a different disease, which were mainly adenocarcinoma and occurred most commonly in females and in patients at the older age [16]. Since the total morbidity of lung cancer was strongly correlated to the age, a possible cause of this type of cancer might be the senescence of immune system [17]. The immune system was considered to be able to kill cancer cells and inhibited tumor growth by innate and adaptive immunity [18]. Our results were consistent with these previous findings. The MEs of midnightblue module and grey60 module were negatively correlated with spontaneous lung cancer susceptibility. It suggested that mouse strains having higher expression of immune system related genes were less possible to have lung cancer.

In the mouse genome, only about 1.5% are proteincoding genes and the majority of transcripts are classified to noncoding RNAs (ncRNAs) [19]. LncRNA is one kind of ncRNAs named by its length (> 200 bp). Partially because of its length, the expression of lncRNA could be detected by mining existing microarray data, although the microarray is not designed for lncRNA analysis. The Affymetrix Exon Array contains more than 5.5 million probes. Its many probes designed for expressed sequence tags (ESTs) and prediction-based transcripts could uniquely map to the lncRNA sequences [14]. Comparing with conducting a new RNA sequencing experiment, it is a relatively cost-effective approach to analyze the lncRNAs by re-proposing the microarray probes for lncRNA expression. In this study, we detected 24,670 lncRNAs expression by re-annotating the microarray probes and 23,333 mRNAs expression at the same time. Thus, we could perform integrative analysis on the interaction between lncRNAs and mRNAs.

With the expression profile of lncRNAs, we constructed a lncRNA co-expression network and found a module significantly correlated with spontaneous lung cancer susceptibility. Totally, 47 lncRNAs were included in this module. To infer the biological function of lncRNAs in spontaneous lung cancer susceptibility, we explored the potential regulatory relationship among lncRNAs in red module and mRNAs in WGCNA process. 40 mRNAs were found to be co-expressed with 30 lncRNAs in red module (|r| > 0.7 and *p* value < 0.01). When we loaded these 40 mRNAs for gene enrichment analysis, similar to lightgreen module in mRNA WGCNA, a loose structure was generated and no significant GO term was found. Meanwhile, among these 40 mRNAs, 26 mRNAs overlapped with genes in lightgreen module in mRNA WGCNA. It inferred that expression of genes in mRNA lightgreen module may be regulated by lncRNAs clustered in lncRNA red module. These genes and lncRNAs were strongly associated with spontaneous lung cancer susceptibility. However, based on the known pathway database, we could not conclude the mechanism in which this mRNA-lncRNA co-expression network involved, and further investigation is needed to explore its effect on spontaneous lung cancer susceptibility.

Using GWA results to filter the mRNA-lncRNA co-expression network, we detected a sub-network including mRNAs (Myo7a and Zfp874a) and two lncRNAs (n290048 and n271850). There were trait-associated SNPs in ± 500k base pairs up- or down-stream of these mRNAs and lncRNAs. Gene Myo7a on chromosome 7 encodes Myosin VIIA. The biological function of Myo7a was mainly found to be associated with Usher Syndrome, Type I [20] and Deafness [21]. No direct association between Myo7a and lung cancer is found. Zfp874a encodes zinc finger protein 874a and there is no literature on relationship between Zfp874a and lung cancer. As a pilot study, the limitation of our project was that no validation experiment of identified genes and lncRNAs was conducted. The biological function of the mRNAs (Myo7a and Zfp874a) and lncRNAs (n290048 and n271850) in lung tissue still requires further investigation.

In summary, we performed a comprehensive system genetic study for spontaneous lung cancer susceptibility. We reported, for the first time, the mRNA/lncRNA modules associated with spontaneous lung cancer susceptibility. Our co-expression network among lncRNAs and mRNAs may provide novel insights of the pathophysiological mechanism of lung cancer and suggest effective targets for lung cancer prevention.

## MATERIALS AND METHODS

### Mice strains and lung tumor susceptibility

In current study, transcriptional data were collected from previous work in our lab [5]. Briefly, transcriptomes were screened in in normal lung tissues from 40 laboratory inbred mouse strains ssing Affymetrix Mouse Exon 1.0 ST microarrays. The strains were 129S1/SvImJ, 129X1/SvJ, A/J, AKR/J, BALB/cByJ, BTBR_T + _tf/J, BUB/BnJ, C3H/HeJ, C57BL/6J, C57BR/cdJ, C57 L/J, C58/J, CAST/EiJ, CBA/J, CE/J, CZECHII/EiJ, DBA/1J, DBA/2J, FVB/NJ, JF1/Ms, KK/HlJ, LG/J, LP/J, MA/MyJ, MOLF/EiJ, MSM/Ms, NOD/LtJ, NON/LtJ, NZB/BlNJ, NZW/LacJ, PERA/EiJ, PL/J, PWD/PhJ, RIIIS/J, SEA/GnJ, SJL/J, SM/J, SPRET/EiJ, SWR/J, and WSB/EiJ. Three female and three male mice were included in each strain. Seven-week old mice were purchased from the Jackson Laboratory (Bar Harbor, ME, USA) and housed for one week in the Washington University Animal Facility. At the age of eight weeks, all mice were killed by cervical dislocation. RNA from lung tissues were isolated for exon array analyses. In this study, all the lung tissues were normal and health.

The data about spontaneous incidence of pulmonary adenomas was collected from an earlier published survey of 28 inbred mouse strains [6]. Briefly, Annerose et al. examined 28 strains of inbred mice for pulmonary adenomas. All investigated mice were obtained, raised, and maintained at the breeding facilities of The Jackson Laboratory (Bar Harbor, ME, USA). Mice were transferred to a specific pathogen-free room at an age of six to eight weeks until they were euthanized by CO_2_ asphyxiation at 20 months (± 28 days) of age. Frequency of pulmonary adenomas were defined by percentage of mice per strain diagnosed as pulmonary adenomas with one or more of the lesions. 23 strains overlapped between this survey and our exon array analyses. We combined our exon array data and the spontaneous incidence of pulmonary adenomas from Annerose’s study of these 23 strain mice to develop the following study.

### RNA data preprocessing and lncRNA re-annotation

For mRNA analyses, all raw CEL files were imported, normalized, background corrected and summarized by the Affymetrix Power Tools (APTs) (http://www.affymetrix.com).

For lncRNA analyses, the microarray data were reannotated using the software noncoder (http://noncoder.mpi-bn.mpg.de/#) [14]. This software was developed for annotating probes that uniquely map to lncRNAs [14]. Briefly, APTs were applied to pre-process the data. Then, the probesets were filtered by the following steps: (1) lncRNAs with less than 3 probes were removed; (2) probes overlapping with protein-coding genes were discarded; and (3) probes were only mapped to known lncRNAs in NONCODE v3.0 [22].

### Weighted gene co-expression network analysis

Network analysis was performed using WGCNA R package [23, 24]. First, we calculated Pearson correlation coefficients for all gene-gene comparisons across microarray samples. Then, the matrix of correlations was converted to an adjacency matrix of connection strengths. The adjacencies were defined as $a_{ij}={\left|cor\left(x_{i},x_{j}\right)\right|}^{\text{β}}$, where *x*_*i*_ and *x*_*j*_ are the i^th^ and j^th^ gene expression traits. The parameter *β* was selected using the scale-free topology criterion previously outlined by Zhang and Horvath [25]. We chose a power of 6, which resulted in an approximate scale-free topology network with the scale-free fitting index R^2^ greater than 0.8. Modules were defined as sets of genes with high topological overlap. The topological overlap measure (TOM) $Tom_{ij}=\frac{\sum_{u\neq i,j}a_{iu}a_{uj}+a_{ij}}{\text{min}\left(k_{i},k_{j}+1-a_{ij}\right)}$, where $\sum u\neq i,ja_{iu}a_{uj}$ denotes the number of nodes to which both *i* and *j* are connected, and u indexes the nodes of the network. A TOM-based dissimilarity measure (1 – TOM) was used for hierarchical clustering. Gene modules corresponded to the branches of the resulting dendogram and were precisely defined using the “dynamic hybrid” branch-cutting algorithm [26]. Module eigengenes (MEs) were defined as the first principal component calculated using PCA, which can summarize modules’ behavior. These MEs were tested for association with spontaneous lung cancer susceptibility by Pearson Correlation. The module membership (MM) (also referred as intramodular connectivity) of gene *i* in module *q*, kME value, was defined as the absolute value of Pearson correlation between its gene expression and the module eigengene. Specifically, $kME_{q}\left(i\right)=\left|cor\left(x_{i},ME_{q}\right)\right|$, where *ME*_*q*_ is the module eigengene of the module *q*. Note that it specifies how close gene *i* is to module *q* and does not require that gene *i* to be a member of the module *q*. MM can be used to find hub genes, which are highly interconnected nodes within gene co-expression modules [23, 27, 28]. The module hub gene was defined as the gene in the module with the highest connectivity or based on a high intra-modular connectivity (MM > 0.75). Similarly, lncRNA genes were also calculated to have the lncRNA modules.

### Gene enrichment analysis

To analyze the potential pathways that traits (spontaneous lung cancer susceptibility) associated modules involved in, we loaded all genes in top four modules (ranked by *p* value) into the Database for Annotation, Visualization and Integrated Discovery (DAVID) [29, 30]. For each module, we examined enrichment of Biological Process gene ontology (GO) terms. The threshold for significant enrichment was FDR < 0.05. The gene-gene interaction networks were drawn via STRING software [31].

### mRNA-lncRNA co-expression network

The mRNA-lncRNA co-expression network was constructed among all mRNAs in WGCNA analysis and lncRNAs in top 1 lncRNA module, ranked by the correlation between modules and spontaneous lung cancer susceptibility. Based on the expression of each mRNA and lncRNA, correlation coefficient and *p* value are obtained for each mRNA-lncRNA pair. The thresholds in this study for filtering were absolute correlation coefficient no less than 0.7 and adjusted *p* value less than 0.01. The filtered mRNA-lncRNA pairs consist the co-expression network.

### GWAS results filtration

The SNP data were obtained from the Mouse HapMap Imputed Genotype Resource (http://mouse.cs.ucla.edu/mousehapmap/), which contained 132k SNPs on commonly used mouse-inbred strains. General Linear Model (GLM) in software TASSEL version 5.0 [32] was used to perform association analysis between SNP and spontaneous lung cancer susceptibility. We used nominal *p* value less than 0.05 as threshold and screened ± 500k base pairs around each mRNA or lncRNA in the mRNA-lncRNA co-expression network.
